# The complete chloroplast genome of *Pleione maculata*, an orchid with important ornamental value and medicinal value

**DOI:** 10.1080/23802359.2021.1948366

**Published:** 2021-07-09

**Authors:** Lie-Fen He, Shao-Juan Qiang, Yong-Hong Zhang

**Affiliations:** School of Life Sciences, Yunnan Normal University, Kunming, China

**Keywords:** *Pleione maculata*, Illumina sequencing, complete chloroplast genome, phylogenetic analysis

## Abstract

*Pleione maculata* is an epiphytic orchid with significant ornamental value and medicinal value. Here, we report the first complete chloroplast genome of *P. maculata*. The circular genome was 158,394 bp in length and consisted of a pair of inverted repeats (IR 26,646 bp), which were separated by a large single copy region (LSC 86,603 bp) and a small single copy region (SSC 18,499 bp). It contained 135 genes, including 89 protein-coding genes, 38 tRNAs and 8 rRNAs. Phylogenetic analysis of cp genomes from 41 species of Orchidaceae revealed that all species of *Pleione* formed one monophyletic clade and *P. maculata* was located at the base of the genus with high bootstrap values (≥99.1%).

The genus *Pleione* D. Don (Orchidaceae), comprising about 33 speices (7 natural hybrid species) of terrestrial, lithophytic or epiphytic orchids, is widely distributed in China, Vietnam, India, Bhutan, Nepal, Thailand, and Myanmar (Chen et al. 2009; Govaerts et al. 2016). *Pleione* are fairly popular ornamental plants in Europe and the USA and widely used as traditional medicine in Asian countries (Teoh 2016). *Pleione bulbocodioides* (Franchet) Rolfe and *P. yunnanensis* (Rolfe) Rolfe are also the original plant of traditional Chinese medicine Shān Cí Gū. The pseudobulb was called commonly ‘ice ball’, which has the functions of clearing heat, detoxifying, and resolving phlegm (You et al. 2011). *Pleione* is also a world-famous ornamental, with showy, color-rich and leafless flowers in flowering period (Zhang et al. 2020).

*Pleione maculata* (Lindley) Lindley & Paxton, distributed in Bhutan, India, Myanmar, Nepal, Thailand and China between 600 and 1600 meters above sea level, is an epiphytic herb growing on tree trunks and mossy rocks in broad-leaved forests (Chen et al. 2009). Pseudobulbs of *P. maculata*, has commonly been applied in the northeast India for the treatment of cuts, wound, or liver complaints (Simpli et al. 2018). In the wild, *P. maculata* is one of the most important parents for a succession of hybrids (Gravendeel et al. 2004; Chen et al. 2009). Although *Pleione* have high ornamental value and medicinal value, there are a few reports on the chloroplast genome. So far, all species with cp genomes reported belong to the Sect. Humiles, such as, *P. bulbocodioides* (Shi et al. 2018), *P. chunii* C. L. Tso (Wu et al. 2019), *P. formosana* Hayata (Jiang et al. 2019), *P. forrestii* Schltr. (Wu et al. 2019) and *P. pleionoides* (Kraenzl. ex Diels) Braem et H. Mohr (Chen et al. 2019). In this study, we assembled and characterized the complete chloroplast of *P. maculata* to provide a better understanding of the phylogeny and genetics of genus *Pleione*.

Fresh leaves of *P. maculata* were collected from the individual growing in the greenhouse of Yunnan Normal University, Kunming, China (24°52'1.41’N, 102°51'19.39’E), and voucher specimen (OP-001) deposited at Herbarium of Yunnan Normal University (YNUB). Total genomic DNA was extracted from fresh leaves using a modified CTAB method (Allen et al. 2006) and sequenced by the Illumina Hiseq 2000 sequencing platform (Illumina, CA, USA) at Novogene (Beijing, China). In total, 3.44 GB of raw data with 11,464,366 raw reads was obtained. Raw reads were filtered by NGS QC Toolkit (Patel et al. 2012). The plastome was de novo assembled using NOVOPlasty (Dierckxsens et al. 2017). After assembled, the genome was automatically annotated using DOGMA (Wyman et al. 2004), then adjusted with Geneious Prime 2020.0.3 (https://www.geneious.com) and submitted to GenBank with accession number MW699846.

The length of the genome sequence of *P. maculata* was 158,394 bp, and it was a typical quadripartite structure. It contained a large single copy (LSC) region (86,603 bp), a small single copy (SSC) region (18,499 bp), and two reverse sequence repeats (IR) regions (26,646 bp). The content of GC in whole chloroplast genome was 37.3%, including 35.2% in LSC region, 30.4% in SSC region and 43.2% in IR region. A total of 135 genes were contained, including 89 protein-coding genes, 38 tRNA genes and 8 rRNA genes. Among them *atp*F, *ndh*A, *ndh*B, *rpl*2, *rpo*C1, *rps*12, *rps*16, *trn*A-UGC, *trn*G-GCC, *trn*I-CAU, *trn*I-GAU, *trn*K-UUU, *trn*L-UAA, *trn*V-UAC contain a single intron, and two introns are contained in *clp*P and *ycf*3.

To clarify the phylogenetic position of *P. maculata*, 41 published chloroplast genomes from Orchidaceae were aligned by using MAFFT 7.308 (Katoh and Standley 2013) with *Goodyera fumata* (GenBank accession KJ501999) and *Ludisia discolor* (GenBank accession NC030540) as outgroups. Maximum-likelihood (ML) tree was constructed using RAxML 8.2.11 (Stamatakis 2014) with the GTR + G nucleotide substitution model. The branch supports were computed with 1000 bootstrap replicates. The phylogenetic tree showed that six species of *Pleione* formed one monophyletic clade with 100% bootstrap value (Figure 1) and *P. maculata* was located at the base of the genus with high bootstrap value (≥99.1%). The complete chloroplast genome of *P. maculata* will provide useful resource for identification, conservation, and utilization of this valuable species. Moreover, it will be helpful to better understand the phylogeny of genus *Pleione* and even the family Orchidaceae.

**Figure 1.**
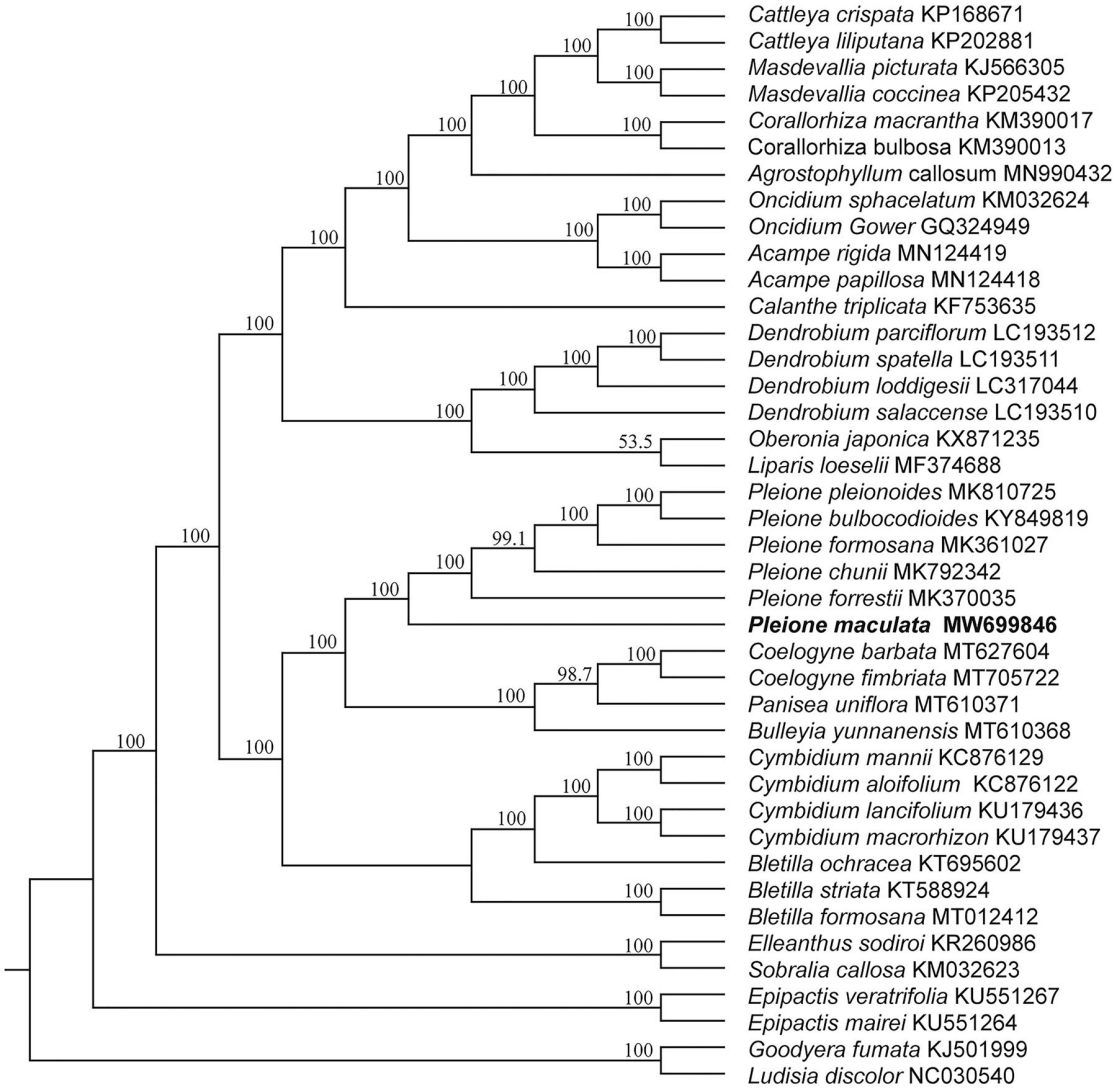
Maximum-likelihood (ML) tree based on 41 complete cp genomes of Orchidaceae with *Goodyera fumata* and *Ludisia discolor* as outgroups. Bootstrap support values are indicated at each branch node and the species, *Pleione maculata*, used in this study is highlighted in bold.

## Data Availability

The genome sequence data that support the findings of this study are openly available in GenBank of NCBI at [https://www.ncbi.nlm.nih.gov/] (https://www.ncbi.nlm.nih.gov/) under the accession number MW699846. The associated BioProject, SRA, and Bio-Sample numbers are PRJNA726024, SRR14354833, and SAMN18918057, respectively.
